# Regulation of alphaherpesvirus protein via post-translational phosphorylation

**DOI:** 10.1186/s13567-022-01115-z

**Published:** 2022-11-17

**Authors:** Tong Zhou, Mingshu Wang, Anchun Cheng, Qiao Yang, Bin Tian, Ying Wu, Renyong Jia, Shun Chen, Mafeng Liu, Xin-Xin Zhao, Xuming Ou, Sai Mao, Di Sun, Shaqiu Zhang, Dekang Zhu, Juan Huang, Qun Gao, Yanling Yu, Ling Zhang

**Affiliations:** 1 Institute of Preventive Veterinary Medicine, Sichuan Agricultural University, Wenjiang, Chengdu, 611130 Sichuan China; 2 Key Laboratory of Animal Disease and Human Health of Sichuan Province, Sichuan Agricultural University, Wenjiang, Chengdu, 611130 Sichuan China; 3 Avian Disease Research Center, College of Veterinary Medicine, Sichuan Agricultural University, Wenjiang, Chengdu, 611130 Sichuan China

**Keywords:** Alphaherpesvirus, viral proteins, protein kinases, phosphorylated proteins, phosphorylation modification

## Abstract

An alphaherpesvirus carries dozens of viral proteins in the envelope, tegument and capsid structure, and each protein plays an indispensable role in virus adsorption, invasion, uncoating and release. After infecting the host, a virus eliminates unfavourable factors via multiple mechanisms to escape or suppress the attack of the host immune system. Post-translational modification of proteins, especially phosphorylation, regulates changes in protein conformation and biological activity through a series of complex mechanisms. Many viruses have evolved mechanisms to leverage host phosphorylation systems to regulate viral protein activity and establish a suitable cellular environment for efficient viral replication and virulence. In this paper, viral protein kinases and the regulation of viral protein function mediated via the phosphorylation of alphaherpesvirus proteins are described. In addition, this paper provides new ideas for further research into the role played by the post-translational modification of viral proteins in the virus life cycle, which will be helpful for understanding the mechanisms of viral infection of a host and may lead to new directions of antiviral treatment.

## Introduction

Herpesviruses constitute a family of DNA double-stranded viruses with a tegument structure. Based on the different biological characteristics and clinical pathogenic characteristics of viruses, the International Committee on the Taxonomy of Viruses (ICTV) categorizes Herpesviridae into three subfamilies: Alphaherpesvirinae, Betaherpesvirinae and Gammaherpesvirinae [1]. For all herpesviruses, a complete virion consists of four parts: a core containing a double-stranded DNA genome, a capsid, an envelope, and a tegument [2]. Alphaherpesvirinae subfamily includes herpes simplex virus type-1/2 (HSV-1/2), duck plague virus (DPV) [3–6], pseudorabies virus (PRV), varicella-zoster virus (VZV), equine herpesvirus (EHV), bovine herpesvirus (BoHV) and Marek’s disease virus (MDV) [7–11].

**Figure 1:**
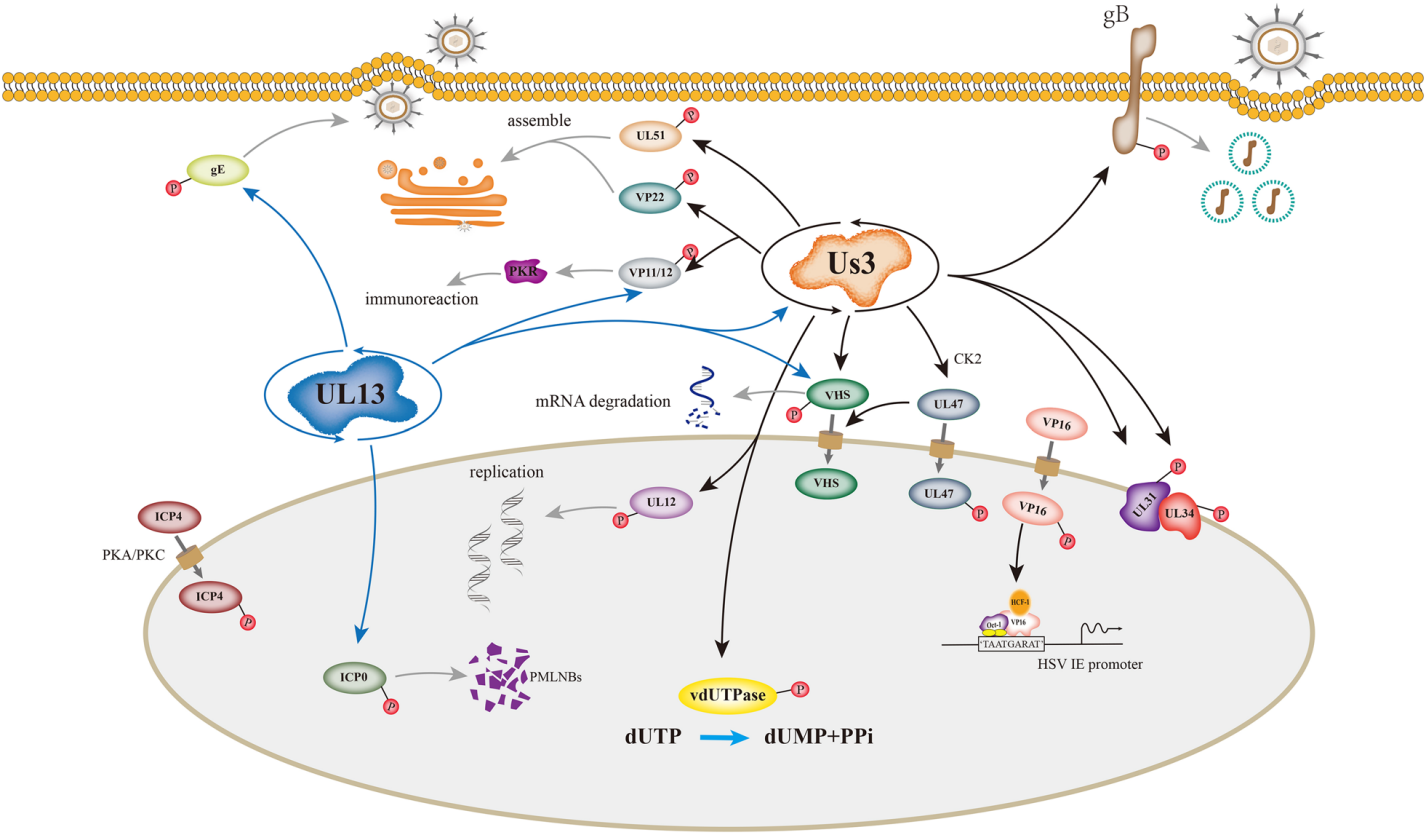
**Summary of the major functions of viral protein kinases and phosphorylated proteins in alphaherpesvirus.** *PKA* protein kinase A, *PKC* protein kinase C, *CK2* Casein kinase 2, *PMLNBs* promyelocytic leukaemia protein nuclear bodies, *HCF-1* host cell factor-1, *Oct-1* octamer-binding transcription factor-1, *IE* immediate early gene.

Protein post-translational modifications, which involve enzyme-mediated covalent addition of functional groups to proteins during or after synthesis, greatly increase the complexity of protein biological functions and result in order-of-magnitude changes between the types of proteins encoded in the genome and their biological functions. Therefore, post-translational modifications greatly expand the coding flexibility of living systems [12, 13]. Post-translational modifications include glycosylation [14, 15], phosphorylation, ubiquitination, methylation, arginylation and acetylation in eukaryotic cells [16–23]. Phosphorylation, in particular, is an important biological process controlled by a series of complex mechanisms to obtain a functional conformation and to regulate organisms exposed to external environmental stimuli and hormone signalling. After infecting host cells, viruses regulate the activity and stability of viral proteins and their interactions with other proteins through phosphorylation and dephosphorylation at various stages of the virus life cycle, thereby participating in a series of viral metabolic activities, such as viral replication and proliferation, as well as the assembly of virus particles (Figure 1). Most phosphorylated proteins carry multiple phosphorylation sites, but this does not mean that all potential phosphorylation sites in a protein molecule can be modified by phosphorylation. Of course, in some cases, phosphorylation at specific sites of certain proteins exerts a great impact on their function. For example, phosphorylation at specific sites in protein kinases regulates their activity [24]. In alphaherpesviruses, many viral protein functions are regulated via phosphorylation. For example, viral protein kinases such as HSV Us3 and UL13 can regulate other protein functions and their own catalytic activity via phosphorylation and autophosphorylation [25, 26]. In particular, the HSV transcriptional regulatory protein ICP4 regulates the underlying genome in sensory neurons through phosphorylation [27]; the HSV glycoprotein B (gB) affects viral replication and pathogenicity in mice through phosphorylation [28, 29]; the HSV-1 tegument protein pUL12 regulates its own nuclease activity through phosphorylation [30, 31]; VP8 of BoHV-1 regulates viral replication and assembly through phosphorylation [32, 33]; and phosphorylation of HSV-1, PRV and VZV pUL51 affects viral replication and pathogenicity [34–36]. In this paper, the effects of alphaherpesvirus protein kinases and the regulatory function of phosphorylation on the function of proteins are summarized to provide new ideas and directions for the future study of alphaherpesviruses.

## Alphaherpesvirus protein kinases

**Figure 2:**
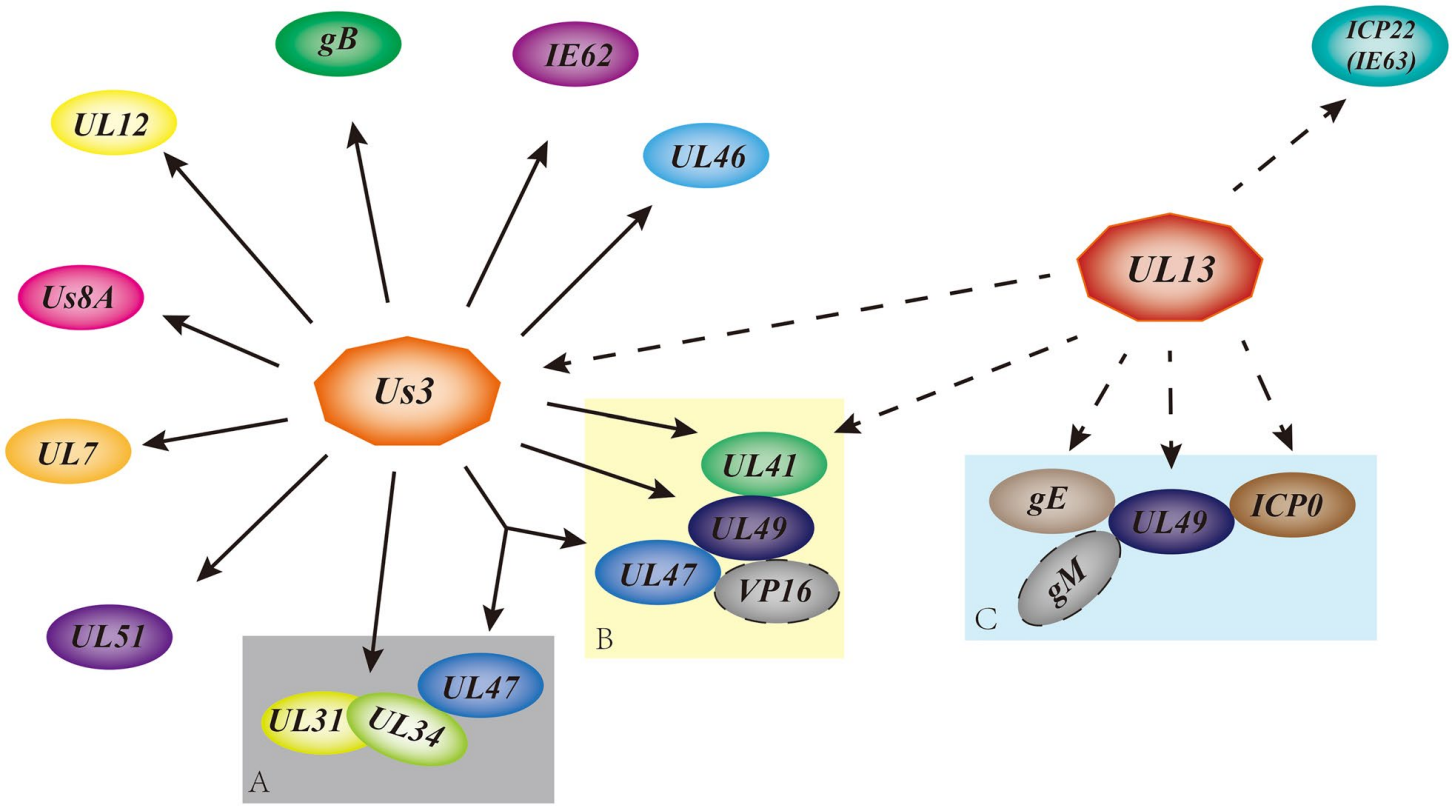
**Interaction networks of phosphorylated proteins in alphaherpesviruses.** The solid line points to a substrate of US3, and the dashed line points to a substrate of UL13. **A**–**C** are phosphorylated proteins that can undergo multiple separate interactions.

The reversible phosphorylation of proteins mediated by protein kinases and phosphatases is one of the most extensively studied post-translational modifications and one of the most common and effective ways in which viruses regulate their protein function [37–39]. In contrast to most viruses, herpesviruses encode specific protein kinases [40], and these protein kinases regulate viral gene expression, nucleocapsid nucleation, viral genome replication, host cell apoptosis, the intracellular transport of viral and cell membrane proteins, and axon transport of capsids to facilitate viral inhibition of the host immune response and achieve effective viral replication [28, 40–51]. In addition, viral protein kinases regulate not only the phosphorylation of viral proteins to regulate their function but also by autophosphorylation, which regulates their catalytic activity [52, 53] (Figure 2). Alphaherpesvirus protein kinases can be classified into the Us3 kinase and its homologues and the UL13 kinase and its homologues.

### Us3 protein kinase and its homologues

Us3 is an early gene encoding a tegument protein that functions similar to protein kinase A (PKA), which is a serine/threonine protein kinase, and is conserved in all alphaherpesviruses but not in other Herpesviridae subfamilies. To date, deletion of Us3 homologue genes in HSV-1, HSV-2, VZV, PRV, MDV-1, BoHV-1/5, or DPV disrupts cell-type-dependent viral replication under cell culture conditions [54–56]. HSV-1 (among the most common of the representative alphaherpesviruses) carries the Us3 kinase, a regulator of viral replication and pathogenicity. Us3 functions related to viral replication and pathogenicity are becoming increasingly clear. For example, Us3 blocks apoptosis induced by viral and cellular proteins [49, 54, 57, 58], regulates the nuclear egress of the nucleocapsid [47, 48, 59, 60], controls the morphology or microtubule network of infected cells [25, 54, 61–63], escapes the host antiviral response [64–67], promotes gene expression through histone deacetylation [68–70] and regulates the intracellular transport of viral and cellular proteins in infected cells [56, 71, 72]. Thus, Us3 is a multifunctional protein that plays various roles in the viral life cycle through the phosphorylation of many viral substrates (Table 1). Moreover, Us3 undergoes self-specific autophosphorylation at Ser147, and Us3 phosphorylated at Ser147 shows higher kinase activity than unphosphorylated Us3 [25]. Although only a small amount of Us3 is autophosphorylated in virus-infected cells, this kinase is required for the proper viral localization and cytopathic induction. In addition, the autophosphorylation of Us3 at Ser147 promotes the development of herpes stromal keratitis (HSK) disease and viral replication in mice [73]. These results indicate that Us3 protein kinase activity is regulated by autophosphorylation at Ser147, mediating viral replication and pathogenicity to a certain extent. The ORF66 protein kinase, a homologue of HSV-1/2 Us3, is a VZV protein kinase originally identified based on its genomic location and homology to other HSV kinases and the presence of classical structural motifs common to all serine/threonine sequences. As mentioned above, protein kinases often maintain kinase function specificity via autophosphorylation; therefore, it is not surprising that ORF66 can be autophosphorylated.

**Table 1:** **Phosphorylation substrates of Us3 homologous protein kinases in alphaherpesvirus**

Alphaherpesvirus	Phosphorylated protein	Phosphorylation site	Functions regulated by phosphorylation
HSV-1/2, PRV, DPV, MDV-1	Us3	Ser-147	Activates kinase activity; promotes HSK development and virus replication [73]
HSV-1	gB	Thr-887	Regulates the expression of gB on the cell surface [28, 29]
HSV-1	Us8A	Ser-61	Increases the neuroinvasive ability of the virus [176, 177]
HSV-2	UL7	Not detected	Induces virus replication and pathogenicity [166]
HSV-1	UL12	Tyr-371, Thr-474 and Ser-604	Regulates exonuclease activity, viral replication, neurovirulence [30, 31, 119]
HSV-1	UL31	Ser-11, Ser-24, Ser-26, Ser-27, Ser-40, and/or Ser-43 [92]	Nuclear membrane localization of UL31/UL34 [47, 92]
HSV-1	UL34	Thr-195, Ser-198	Not reported
HSV-1	UL41 (VHS)	Not detected	Degrade host mRNAs, shut down host protein synthesis; induce viral replication and regulation of the PKR-mediated immune response [171]
HSV-1	UL46 (VP11/12)	Not detected	Induces expression of viral genes, interacts with other proteins and viral progeny s [178, 180]
HSV-1, BoHV-1	UL47	Ser-77,Ser-88 and Thr-685	Induces viral DNA encapsulation and secondary envelopment [33] and nuclear localization [71, 136]
BoHV-1	UL49(VP22)	Not detected	Induce virus assembly [149, 150, 153]
HSV-1	UL51	Ser-184	Induces viral replication and virulence [34]
VZV	IE62	Ser-686, Ser-722	Promotes cellular localization [161, 162]

### UL13 protein kinase and its homologues

Similar to the Us3 protein kinase, the UL13 protein is a serine/threonine protein kinase located in the tegument structure [74]. HSV-1/2, DPV, MDV, PRV UL13 and VZV ORF47 are homologous [75], and the HSV-1 and HSV-2 UL13 homologues show the most similar amino acid sequence homology, which is 85% identical [76]. The widespread presence of UL13 also suggests that UL13 plays an important role in the life cycle of alphaherpesviruses.

Overall, UL13 exhibits three functions. First, UL13 promotes the disintegration of virions via the phosphorylation of other tegument proteins in the early stage of infection [77] and regulates the expression of viral genes through its kinase activity, such as by regulating ICP22 activity [43], stabilizing and regulating ICP0 activity [78, 79], and regulating VP11/12 activity [80]. Second, UL13 can inhibit interferon signalling pathway activation [81–83] and the transcription of host genes via the phosphorylation of cytokines [84]. Finally, UL13 can affect the horizontal transmission of a virus [85] (Table 2). Both HSV-1 UL13 and HSV-2 UL13 have been reported to be autophosphorylated in vitro [78, 86, 87]. Ser-118 and Ser-121 of HSV-2 UL13 were replaced with alanine residues, and both of the mutations significantly reduced the autophosphorylation of UL13. However, only the mutant in which Ser-121 was replaced with alanine significantly reduced the ability of HSV-2 UL13 to phosphorylate exogenous substrates. Similarly, Koyanagi et al. [26] found that phosphorylation of HSV-2 UL13 Ser-18 regulated its function in infected cells and that this regulatory effect was critical for HSV-2 replication and pathogenesis in vivo. These results indicate that Ser-18, Ser-118 and Ser-121 are the sites of UL13 autophosphorylation or are important to the recognition motif and that the autophosphorylation of HSV-2 UL13 affects the ability of this kinase to phosphorylate exogenous substrates, which is not strictly dependent on autophosphorylation [87]. ORF47, another serine/threonine kinase in VZV, is a homologue of the HSV UL13 protein kinase and an important regulator of the pathogenesis of VZV. Similar to UL13, ORF47 is autophosphorylated, but autophosphorylation is not required for its catalytic activity [88, 89].

**Table 2:** **Phosphorylation substrates of UL13 homologous protein kinases in alphaherpesvirus**

Alphaherpesvirus	Phosphorylated protein	Phosphorylation site	Function of phosphorylated proteins
HSV-1/2, VZV	UL13	Ser-18, Ser-118 and Ser-121	Activates kinase activity [87]
HSV-1	Us3	Residues 405 to 481	Not reported
VZV	gE	Residues 590 to 602	Induces viral release and cell-to-cell spread [172]
HSV-1, VZV	ICP22(IE63)	Not detected	Stabilizes or increases the transcription of a specific subset of viral RNAs [43]
HSV-1	ICP0	Ser-224, Thr-226, Thr-232, Thr-232, Ser-365, Ser-367, Ser-371, Ser-508, Ser-514, Ser-517 and Thr-518	Affects the subcellular and subnuclear localization of ICP0; shows trans-activating activity and immune escape [110]
HSV-1	UL41(VHS)	Not detected	Table 1
HSV-1	UL46(VP11/12)	Not detected	Table 1
HSV-1/2, VZV	UL49(VP22)	Not detected	Table 1

### Synergistic effect of the protein kinases Us3 and UL13

In all Herpesviridae, protein kinases encoded by viruses can be classified into two main categories, namely, conserved herpesvirus protein kinases (CHPKs) are conserved in the α-, β-, and γ-Herpesviridae subfamilies [90], and the remaining protein kinases are found only in the α-Herpesviridae subfamily [55]. UL13 is a CHPK, but Us3 is an α-subfamily kinase. It has been reported that Us3 kinase deletion did not affect viral DNA accumulation or virion-release levels but resulted in nucleocapsid aggregation, which was strictly cell type dependent [59, 91–93]. Knocking out UL13 resulted in a significant reduction in extracellular virions (almost a 30-fold decrease at a low MOI). This effect was greatly enhanced when both Us3 and UL13 kinases were knocked out [94], suggesting that UL13 may functionally complement the kinase activity of Us3 and that HSV-1 protein kinases promote viral replication in a cooperative manner comparable to CHPKs in β- and γ-herpesviruses during viral replication. In addition, Akihisa [95] demonstrated that UL13 specifically and directly phosphorylated the Us3 peptide encoded by a codon sequence from position 405 to 481 in vitro, and UL13-mediated phosphorylation of Us3 was not necessary for optimal kinase activity of Us3 in infected cells. In conclusion, UL13 functionally supplements Us3 kinase activity in vivo, but this effect is not induced by phosphorylation.

## Alphaherpesvirus phosphorylated proteins

### Phosphorylation of the serine-rich region of ICP4

ICP4 is an immediate early gene of HSV that encodes the main transcriptional regulatory protein of this virus [96, 97], which is necessary for the early and late transcription of viral genes and the inhibition of its own expression and that of other immediate early genes of the virus [98–100]. In infected cells, ICP4 has been shown to be phosphorylated at serine and threonine residues [101, 102], and the serine-rich region (residues 142–210) of ICP4 has been identified as the target of phosphorylation, with multiple serine residues and one threonine residue phosphorylated [103]. Previous reports have led to a hypothesis suggesting that after viral infection, ICP4 is first phosphorylated by PKA, PKC, or another kinase at its serine-rich region, and then, phosphorylated ICP4 stimulates ICP4-related or shows inherent kinase activity, which triggers the phosphorylation in the remainder of the ICP4 molecule in either a cis or trans manner. Thus, ICP4 may be continuously phosphorylated at multiple phosphorylation sites [103, 104]. In addition, the serine-rich region of ICP4 can induce ICP4 phosphorylation and viral replication, but this region is not absolutely necessary for this effect; therefore, certain ICP4 phosphorylation mechanisms function independent of its serine-rich region [103]. Kramer et al. [27] found that ICP4 is expressed and phosphorylated at low levels during latent infection, which may affect the latent state by inhibiting its activity. Phosphorylation of ICP4 may affect its ability to bind DNA and inhibit transcription. Thus, phosphorylation of ICP4 regulates underlying gene expression in sensory neurons via a signal transduction pathway.

### Phosphorylation of the three major phosphorylation regions (11 potential phosphorylation sites) in ICP0

ICP0 is a 110 kDa phosphorylated nuclear protein that transactivates the early and late genes of HSV-1 as well as many cellular and other viral genes [105, 106]. In addition, ICP0 is a SUMO-targeted ubiquitin ligase (STUbL). Its significant function is induction of promyelocytic leukaemia protein nuclear body (PML NB) degradation through the ubiquitin protease pathway [107]. To date, two kinases have been confirmed to affect the phosphorylation of ICP0: Cdk1 phosphorylates the second exon (residues from 20 to 241) of ICP0 in vitro [108], Cdk2 phosphorylates the SUMO-binding motif SLS4 in ICP0, which increases the affinity of ICP0 for ubiquitinated proteins and enhances the STUbL activity of ICP0. In addition, ICP0 can neutralize the host antiviral response via phosphorylation of SLS4 mediated by Cdk2 to affect the degradation of PML NBs [109]. Davido et al. showed that ICP0 carries at least three phosphorylation-prone regions (11 potential phosphorylation sites): Phos.1 (Ser-224, Thr-226, Thr-232 and Thr-232), Phos.2 (Ser-365, Ser-367 and Ser-371) and Phos.3 (Ser-508, Ser-514, Ser-517 and Thr-518) [110]. Mutations in these three phosphorylation regions affect subcellular and subnuclear localization of ICP0 (Phos.3), ND10-disrupting activity in certain cells (Phos.2 and Phos.3), and transactivation activity in Vero cells (Phos.1 and Phos.3). Among these mutants, Phos.1 exerts the greatest influence on ICP0-induced transactivation and viral replication [110]. Thus, phosphorylation of ICP0 affects not only its subcellular localization but also its transactivation activity and antiviral response to host cells.

### Phosphorylation of gB at Thr-887

In the herpesvirus family, gB is a glycoprotein that is highly conserved in the envelope, which allows the virus to adsorb onto a host cell plasma membrane, mediates the fusion of the virus envelope and cell membrane, allows viral penetration and intercellular diffusion, and causes viral cyclic replication [111–113]. HSV-1 gB is a substrate of Us3, and its phosphorylation site, Thr-887, is located near an endocytic motif. After replacing the residue at this site with alanine, the endocytosis of gB was significantly increased, thus downregulating the cell surface expression of gB in infected cells. However, the wild-type phenotype was restored after a phosphorylation mutation of this site was simulated [28, 29]. gB on the cell surface is an important target for inducing antibody-dependent cytotoxicity and an effective inducer of the immune responses in vivo [114–116]. The lysis of HSV-1-infected cells by natural killer cells is related to the amount of gB on the cell surface [114]. Therefore, phosphorylation of gB by Us3 reduces the amount of gB molecules on the cell surface exposed to the immune system, which may make it difficult for the infected cells to be recognized and attacked by the immune system in vivo. Consistent with this supposition, blocking the phosphorylation of gB at Thr-887 significantly reduced the development of HSK disease and viral replication in mice with ocular infection. These results suggest that phosphorylation of HSV-1 gB at Thr-887 plays an important role in viral replication and pathogenicity in mice by regulating the expression of gB on the cell surface and nucleation of the nucleocapsid.

### Phosphorylation of pUL12 at Thr-371, Thr-474 and Ser-604

The HSV-1 UL12 gene encodes an alkaline nuclease with 5′–3′ exonuclease activity and is homologous to nucleases of other viruses in the Herpesviridae family [117, 118]. The exonuclease activity of pUL12 is necessary for HSV-1 to generate infectious viral particles that spread between cells, and the phosphorylation of pUL12 regulates nuclease activity and plays an important role in viral replication and pathogenesis [30, 31, 119]. HSV-1 pUL12 has been reported to carry three phosphorylation sites: Tyr-371, Thr-474 and Ser-604. Among these sites, Tyr-371 is highly conserved in pUL12 homologues in all herpesvirus subfamilies, and the regulation of phosphorylation at this site in the pUL12 protein has been investigated via mutation of Tyr-371. The results of mutation showed that the phosphorylation of Tyr-371 remained unaffected, of the expression of pUL12 stable and pUL12 exonuclease activity in HSV-1-infected cells proceeded in a cell-dependent manner; this effect was most obvious in HEL cells [31]. In addition, phosphorylation of Tyr-371 was required for the efficient replication of HSV-1 in cell culture, and its effect on viral replication varied with cell type and MOI. Similarly, intracranial injection of an pUL12 Tyr-371 mutant virus led to much lower neurotoxicity in mice than that the injection of a mutant virus that was pseudophosphorylated at this site [31]. These results suggest that pUL12 mainly promotes HSV-1 replication, intercellular diffusion and neurotoxicity in mice through the phosphorylation of Tyr371.

### Phosphorylation of the nucleus-egressing complex UL31/UL34

In recent years, studies on HSV-1, HSV-2, PRV, EHV-1 and DPV have shown that the UL31 protein plays a positive role in the formation of the primary envelope of the viral nucleocapsid and the synthesis, assembly and release of viral DNA and that it regulates the DNA damage response of virus-infected cells [120–125]. The HSV-1 UL31 protein is an alkaline phosphorylated nuclear protein with a hydrophilic amino terminal and nuclear localization signal sequence [126]. HSV-1/2 UL34 is a membrane-anchoring protein that can also function as a nucleoplasmic protein. It affects the redistribution of the endoplasmic reticulum (ER) around the nuclear membrane and is necessary for the effective localization of ER-associated regulators related to the nuclear egress of a nucleocapsid to the nuclear membrane [127, 128]. UL31 and UL34 proteins interact with each other in the nuclear membrane to form a nuclear egress complex (NEC), enhancing the stability of each other. Removal of one protein leads to the mislocalization of the other protein, and NEC plays an important role in the formation of the primary envelope of a virus [129–131]. Serine phosphorylation of HSV-1 UL31 by US3 has been reported to be essential for the function of the UL31 protein, which is involved in regulating the proper localization of UL31/UL34, the formation of the primary envelope, and the fusion of the nascent virion envelope with the outer nuclear membrane [47, 48, 92, 95]. After the deletion of Us3, UL31 and UL34 accumulated abnormally in the nuclear membrane, presenting as distributed puncta and leading to nuclear invagination with multiple primary envelope virions. However, in the absence of Us3 protein kinase activity, a pseudophosphorylated UL31 mutant of UL31 restored the wild-type localization of UL31 and UL34, suggesting that Us3 regulates the localization of UL31 and UL34 to the nuclear membrane through phosphorylation of UL31 [62, 132–134]. Nevertheless, phosphorylation of UL34 Thr-195 and Ser-198 by US3 exerted little effect on viral replication, localization of UL34 and UL31, or their nuclear export [48]; therefore, although UL34 was phosphorylated, the significance of its phosphorylation was unclear. In addition, phosphorylation of PRV UL31, a protein homologous to HSV-1 UL31, may play an important role during PRV infection, possibly by regulating the proper localization of the virus or other uncharacterized functions; for example, it may play regulatory roles in NEC localization and nucleocapsid expulsion from a cell [135].

### Phosphorylation of pUL47 at Ser-77, Ser-88 and Thr-685

VP8, a 96 kDa phosphorylated protein encoded by the UL47 gene, is one of the most abundant tegument proteins in BoHV-1 and is critical for viral replication. Casein kinase 2 (CK2) and Us3 mediate BoHV-1 post-translational phosphorylation [33]. By mutating the VP8 protein of BoHV-1, Zhang et al. [33] found that the effect of VP8 on virions mainly occurred in two stages: one stage before or during the expulsion of the capsid, during which no phosphorylation is needed, and the other stage is in the Golgi apparatus, where phosphorylation of VP8 is needed. This finding indicates that phosphorylation of VP8 plays a role in DNA encapsulation and secondary envelopment of the virus, while nuclear localization of VP8 is not affected by phosphorylation. In contrast, NLS-mediated nuclear localization of VP13/14 (a homologue of VP8) in HSV requires Us3 phosphorylation of VP13/14 [136], which temporally occurs between cell penetration and viral protein synthesis [77]. Kato et al. conducted biological analysis of the phosphorylation sites of pUL47 and found that the phosphorylation sites of pUL47 (Ser-77, Ser-88 and Thr-685) were all very close to the nuclear localization signal (NLS) sequence and nuclear export signal (NES) sequence. It has been reported that the sites near the phosphorylated protein NLS sequences are common regulatory sites that mediate the transport of NLS-containing proteins into the nucleus. Therefore, it can be speculated that the phosphorylation of pUL47 regulates its nuclear localization in cells. Consistent with this supposition, a Ser-77 mutation in pUL47 affected the nuclear localization of pUL47 in cells, and after pseudophosphorylation of this mutated site, pUL47 showed wild-type nuclear localization [71]. These results suggest that phosphorylation of pUL47 promotes nuclear exportation of the virus by regulating its nuclear localization in infected cells.

### Phosphorylation of VP16 at Ser-355 and Ser-375

VP16, a phosphorylated protein encoded by the UL48 gene, is a transcriptional activator of immediate early genes. In alphaherpesviruses, homologous VP16 proteins carry transactivation domains (TADs) and DNA-binding domains (DBDs), which are called core domains. The DBD of the VP16 protein is conserved in multiple alphaherpeviruses, but neither the location nor sequence of the TAD is conserved [137]. In the case of HSV, once a virus infects a host cell, VP16 forms a transcription regulatory complex with two cellular proteins, octamer-binding transcription factor 1 (Oct-1) and host cell factor-1 (HCF-1), to activate the transcription of immediate early genes [138–141]. The phosphorylation of VP16 mainly occurs at a serine residue, with threonine phosphorylation rare, and tyrosine phosphorylation negligible. VP16 can be phosphorylated by one or more cellular kinases, and the main phosphorylation sites are located at C-terminal serine residues downstream of amino acid 370 [142, 143]. Phosphorylation of HSV VP16 at Ser-355 and Ser-375 regulates the interaction of VP16 with HCF-1 and Oct-1, which is required for the Oct-1-HCF-1-VP16 complex to bind to an immediate early gene promoter [144]. In summary, phosphorylation of VP16 switches on the VP16 transcriptional activation function and regulates the earliest stage of HSV entry into the lytic cycle and in vivo latent reactivation.

### Phosphorylation of the tegument protein VP22

VP22 is a 300-amino acid structural protein encoded by the UL49 gene in herpesviruses. It is one of the most abundant tegument proteins, with more than 2000 copies per virion [145]. VP22 can interact with a variety of tegument proteins and glycoproteins to regulate virion assembly, mediate the transport of capsid proteins from the outer membrane of the nucleus to the Golgi apparatus, and promote the formation of mature virions [146]. In addition, the VP22 proteins of HSV-1, HSV-2, VZV and BoHV-1 can be post-translationally modified via phosphorylation. Moreover, during HSV-1/2 infection, the tegument protein VP22 exists in both phosphorylated and nonphosphorylated forms, and only the VP22 with low phosphorylation levels is assembled into an virion. However, phosphorylation is not required for efficient assembly into the tegument [147–152], suggesting that the phosphorylation levels of VP22 regulate the assembly of virus particles. Similarly, a tyrosine phosphorylation site in BoHV-1 VP22 is critical for the effective assembly of VP22 into a virion [149, 150, 153]. Many viruses encode their own kinases; therefore, the kinases critical for VP22 phosphorylation vary among herpesviruses. For example, CK2 and UL13 are protein kinases critical for phosphorylating VP22 in HSV-1-infected cells. In VZV, ORF47 is the protein kinase critical for the phosphorylation of VP22, while in BoHV-1, Us3 is the protein kinase critical for phosphorylation of VP22 [32, 150, 151, 153].

### Phosphorylation of pUL51 at Ser-184

The HSV-1 pUL51 protein is conserved in the α-, β- and γ-herpesvirus subfamilies. It is phosphorylated by protein kinases and plays an important role in viral secondary envelopment and cell-to-cell spread, as well as in the effective replication of the virus in cells [34–36, 154–158]. Akihisa et al. identified a functional phosphorylation site in pUL51, thereby elucidating the regulatory mechanism of pUL51 in HSV-1 infected cells [34]. They identified five phosphorylation sites of pUL51 via mass spectrometry and found that mutations in the phosphorylation site Ser-184 induced abnormal accumulation of primary enveloped virions in the nuclear membrane space and accumulation of secondary enveloped virions in the cytoplasm. Moreover, Ser-184 phosphorylation significantly affected the virulence of HSV-1. These results indicate that phosphorylation of pUL51 is crucial for morphological changes and the stable local adhesion of HSV-1-infected cells, as well as for the replication and pathogenicity of the HSV-1 virus itself.

### Phosphorylation of IE62 at Ser686 and Ser722

IE62 is a nuclear transcriptional regulatory protein of VZV that drives VZV transcription through interactions with transcriptional activators and components of the mediator complex [159, 160]. It was reported that IE62 was distributed in the nucleus at the early stage of VZV infection before the expression of the ORF66 protein kinase. With increasing expression of the ORF66 protein kinase, IE62 begins to accumulate in the cytoplasm. However, in VZV-infected cells without ORF66 kinase, IE62 no longer appears in the cytoplasm. The kinase activity of ORF66 is necessary for the localization of IE62 in cells. In addition, ORF66 directly phosphorylates the Ser-686 and Ser-722 sites of IE62 in cells and in vitro [161, 162]. Moreover, Ser-686 is highly conserved in all IE62 homologues of herpesviruses, and this site is closely associated with a nuclear input signal, again suggesting that the cellular localization of viral proteins may be regulated by their phosphorylation. In addition to OFR66, IE62 is phosphorylated by the ORF47 kinase and CK2 [163]. The VZV protein phosphorylated by ORF47 is a homologue of HSV ICP22, IE63, which is a major transcript found in the human ganglion and latent animal models of VZV infection [164, 165].

In addition to the aforementioned proteins, many other viral proteins can be phosphorylated, which regulates their functions. For example, UL7 is phosphorylated by HSV-2 Us3. Although its phosphorylation exerted no effect on viral replication in cell culture, it was necessary for effective replication and pathogenicity of HSV-2 in vivo [166]; ICP1/2, also known as VP1/2, is a 270 kDa structural protein located in the HSV-1 tegument that is prone to serine phosphorylation, but the role played by phosphorylated VP1/2 has not been clarified [167]. The virion host shutoff (VHS) protein encoded by HSV UL41 specifically degrades mRNA, inducing the abrogation of host gene expression. Phosphorylation of VHS by Us3 and UL13 regulates the degradation of host mRNAs and the abrogation of host protein synthesis, contributing to the effective replication of the virus and regulating the protein kinase R (PKR)-mediated immune response [168–171]; VZV gE is phosphorylated by serine/threonine protein kinases and CK2 in mammalian cells [172, 173], while Us8A is colocalized with gE in HSV-1 and is phosphorylated in infected cells [174, 175]. Us8A Ser-61 is a target of Us3, and Us3 likely regulates the nerve invasion ability of the virus by regulating phosphorylation at Ser-61, particularly enhancing the replication ability of the virus in the trigeminal ganglion, further suggesting that phosphorylation of Us8A Ser-61 can effectively regulate viral replication [176, 177]. In addition, HSV VP11/12 is not only phosphorylated by viral protein kinases Us3/UL13 or cell protein kinases on serine/threonine residues but also phosphorylated on tyrosine residues. This tyrosine phosphorylation modification may help regulate the expression of viral genes in lymphocytes and alter their interactions with other tegument proteins or progeny viruses during assembly [77, 178–180].

## Multiple interactions between phosphorylated alphaherpesvirus proteins

### Interaction among VHS, VP22, VP16, and pUL47

VHS is an endoribonuclease that degrades cell and viral mRNA. The degradation of cellular mRNA by VHS promotes the synthesis of viral proteins by increasing the availability of cellular translation mechanisms, while the degradation of viral mRNA helps regulate the sequence expression of different viral genes [181–184]. In the course of viral infection, many viral proteins cooperate in the regulation of VHS activity to optimize the proliferation of viruses in host cells. For example, VP22 can promote the accumulation and translation efficiency of viral mRNA in the late stage of infection and can increase mRNA abundance by protecting mRNA from degradation through its RNA-binding activity [185]. The absence of VP22 leads to a significant reduction in translation efficiency, but this effect does not depend on mRNA abundance, and secondary mutations in VHS compensate for translation defects, suggesting that in HSV-1 infection, VP22 plays a role in regulating the translation inhibitory activity of VHS [186]. In addition, as a part of the VP22-VP16-VHS complex, the VHS protein can interact with VP16-VP22 complex during the initiation of translation but cannot interact with VP22 alone; therefore, VP22 and VP16 jointly regulate the activity of VHS [187, 188]. Finally, pUL47 has been reported to deliver a portion of VHS to the nucleus. In transfected cells, NES-mutated VHS degraded stable mRNA, but not in infected cells, suggesting that pUL47 largely blocks VHS degradation of viral mRNA [189]. In summary, the VP22, VP16 and pUL47 proteins regulate intracellular VHS levels, regulate viral gene cascade activation mechanisms and maintain viral mRNA degradation activity.

### Interaction among pUL47, UL31, UL34, and Us3

It has previously been reported that pUL47 can form a complex with Us3 in HSV-1-infected cells, and pUL47 can colocalize with UL31 and UL34 to the nuclear membrane regardless of whether Us3 shows catalytic activity [71], increasing the likelihood that pUL47 interacts with UL31, UL34 and Us3 on the nuclear membrane. Similarly, Liu et al. [190] found that pUL47, UL31, UL34 and Us3 interacted on the nuclear membrane in a immunoprecipitation experiment. After the kinase activity of Us3 was eliminated, pUL47, UL31 and UL34 were all abnormally located in the nuclear membrane, presenting as puncta [190]. These results indicate that in HSV-1-infected cells, the localization of pUL47, UL31 and UL34 on the nuclear membrane is regulated by Us3 kinase activity, and the four proteins can jointly localize to the nuclear membrane to form higher-order complexes. In addition, loss of pUL47 resulted in aggregation of the capsid in the nucleus and a significant decrease in the number of primary envelope virions in the nuclear space. Thus, pUL47 promotes the formation of the HSV-1 primary envelope via its regulatory function mediated by its interactions with the UL31/UL34 complex and Us3, key regulators of HSV-1 nuclear egress.

### Interaction among gE,VP22, gM, andICP0

Elliott et al. [191] found that HSV VP22 interacted with the immediate early protein ICP0, which not only affected ICP0 expression, subcellular localization and its participation in viral assembly but also inhibited the transactivation ability of ICP0 [192]. The absence of VP22 led to cell type-specific replication defects in MDBK cells and delays in viral protein synthesis, especially the production of the immediate early protein ICP0. Further studies have shown that this effect of VP22 on ICP0 is caused by ICP0 phosphorylation [152, 191]. A gE-VP22-gM-ICP0 multicomponent glycoprotein-tegument complex has been identified in HSV-1-infected cells. The complex centres on VP22, and its C-terminal binds to gE and gM, while its N-terminal recruits ICP0 to the complex [193]. VP22-ICP0 can form, but gE and gM are both needed for the multicomponent complex to form effectively and reside stably in a cell, suggesting that gE and gM may stabilize the binding of ICP0 through VP22 [193]. Interestingly, the glycoprotein-binding domain of VP22 is conserved in all alphaherpesviruses [194], suggesting that the gE-VP22-gM(-ICP0) complex may be critical for the replication of alphaherpesviruses.

## Conclusion

After a virus infects host cells, a number of mechanisms are activated to induce or inhibit the cascade of intracellular signalling pathway activation, through phosphorylation and dephosphorylation to regulate the activity and stability of viral proteins and their interactions with intracellular proteins; their regulatory effects of viral replication, virus proliferation and virus particle assembly; and a series of metabolic activities. For example, major structural components of the HSV-1 tegument are phosphorylated when the virus enters an infected cell, and this phosphorylation mediates the dissociation of the tegument from the capsid in vivo under the action of VP13/14 and VP22 [77]. In addition, some viral proteins, such as VP1/2, VP13/14, VP16 and VP22, are phosphorylated during virion assembly and dephosphorylated after virion maturation, suggesting that phosphorylation and dephosphorylation are mechanisms that regulate HSV-1 dissociation and assembly. In addition to regulating the performance and metabolism of the viruses themselves, the evolution of phosphorylation has driven phenotypic diversity among species, further influencing their adaptation during evolution [166]. From this perspective, the effect of viral protein phosphorylation on antiviral drugs effectivity is very helpful for treatment development, but not all of the viral protein phosphorylation events during physiological viral activities show a significant regulatory effect; therefore, further study of viruses in host cell protein phosphorylation and their corresponding regulatory functions will lead to a better understanding of the viral infection mechanisms and identification of effective targets for virus prevention and control.
